# Thorough Characterization of *ETHQB3.5*, a QTL Involved in Melon Fruit Climacteric Behavior and Aroma Volatile Composition

**DOI:** 10.3390/foods12020376

**Published:** 2023-01-13

**Authors:** Noelia Dos-Santos, María C. Bueso, Aurora Díaz, Eduard Moreno, Jordi Garcia-Mas, Antonio J. Monforte, Juan Pablo Fernández-Trujillo

**Affiliations:** 1 Department of Agronomical Engineering, Technical University of Cartagena (UPCT), Paseo Alfonso XIII, 48. ETSIA, E-30203 Cartagena, Spain; 2 Department of Applied Mathematics and Statistics, UPCT-Technical University of Cartagena, Doctor Fleming s/n. ETSII, E-30202 Cartagena, Spain; 3 Department of Plant Sciences, Agrifood Research and Technology Centre of Aragon (CITA), Avda. Montañana 930, E-50059 Zaragoza, Spain; 4 AgriFood Institute of Aragon—IA2, CITA-University of Zaragoza, E-50059 Zaragoza, Spain; 5 IBMCP Instituto de Biología Molecular y Celular de Plantas, CSIC/Universidad Politécnica de València, Ciudad Politécnica de la Innovación—Edificio 8E Ingeniero Fausto Elio, s/n, E-46022 València, Spain; 6 Center for Research in Agricultural Genomics (CRAG) CSIC-IRTA-UAB-UB, Edifici CRAG, Campus Universitat Autònoma de Barcelona (UAB), Bellaterra, E-08193 Barcelona, Spain; 7 Institut de Recerca i Tecnologia Agroalimentàries (IRTA), Edifici CRAG, Campus UAB, Bellaterra, E-08193 Barcelona, Spain; 8 Institute of Plant Biotechnology, UPCT-Technical University of Cartagena, Plaza del Hospital s/n, Campus Muralla del Mar, E-30202 Cartagena, Spain

**Keywords:** candidate genes, *Cucumis melo* L., ethylene production, fruit quality, near-isogenic lines, respiration rate, quantitative trait loci, volatile organic compounds

## Abstract

The effect of the QTL involved in climacteric ripening *ETHQB3.5* on the fruit VOC composition was studied using a set of Near-Isogenic Lines (NILs) containing overlapping introgressions from the Korean accession PI 16375 on the chromosome 3 in the climacteric ‘Piel de Sapo’ (PS) genetic background. *ETHQB3.5* was mapped in an interval of 1.24 Mb that contained a NAC transcription factor. NIL fruits also showed differences in VOC composition belonging to acetate esters, non-acetate esters, and sulfur-derived families. Cosegregation of VOC composition (23 out of 48 total QTLs were mapped) and climacteric ripening was observed, suggesting a pleiotropic effect of *ETHQB3.5*. On the other hand, other VOCs (mainly alkanes, aldehydes, and ketones) showed a pattern of variation independent of *ETHQB3.5* effects, indicating the presence of other genes controlling non-climacteric ripening VOCs. Network correlation analysis and hierarchical clustering found groups of highly correlated compounds and confirmed the involvement of the climacteric differences in compound classes and VOC differences. The modification of melon VOCs may be achieved with or without interfering with its physiological behavior, but it is likely that high relative concentrations of some type of ethylene-dependent esters could be achieved in climacteric cultivars.

## 1. Introduction

Genetic dissection of fruit aroma volatiles is difficult because of the polygenic nature of these traits, which remains largely unknown [1]. Most of the Quantitative Trait Loci (QTLs) associated with volatile organic compounds (VOCs) have been mapped in climacteric fruits, such as apples [2], peaches [3,4,5], melons [6,7,8], or tomatoes [1,9,10,11,12,13], but also in non-climacteric ones such as grapes [14,15], strawberries [16], and raspberries [17]. One of the difficulties encountered in the genetic dissection of VOCs is the huge number of compounds involved and the difficulties associated with their analysis and quantification [18,19,20]. Multivariate statistical tools, including correlation network analysis or clustering procedures, are useful for identifying interactions between VOCs and for grouping highly correlated compounds, allowing dimensionality reduction [20,21].

Melon germplasm displays a very high degree of genetic variability underlying its molecular, morphological, physiological, and compositional characters at plant and fruit levels, thus providing a reservoir of genetic variability for improving melon cultivars [22,23,24,25,26,27,28], although only a small part has been mapped in integrated maps [25].

Melons have been proposed as alternative models to tomatoes for studying climacteric fruit ripening because of the presence of climacteric and non-climacteric cultivars within the same species [29]. In the first attempt to decipher the genetic control of climacteric ripening, Périn et al. [30] described two major genes (*Al-3* and *Al-4*, located on chromosomes 8 and 9, respectively) as being responsible for the genetic control of climacteric ripening, together with other QTLs located in different chromosomes with lesser effects. Then, QTLs with a major effect on climacteric ripening have been mapped in different experimental populations [31,32,33,34,35,36]. Among them, *ETHQB3.5* was identified in the Near-Isogenic Line (NIL) SC3-5 that carries an introgression on chromosome 3 from the Korean accession PI 16375, cultivar Shongwan Charmi (SC), into the Spanish ‘Piel de Sapo’ (PS) genetic background [31,37]. Remarkably, both PS and SC are non-climacteric cultivars [38]. Additionally, another set of NILs with shorter introgressions in the same region of chromosome 3 from the same cross has been used to study the possible effects of *ETHQB3.5* on traits of interest for postharvest purposes, including the retention of certain flavors following cold storage [39] or the production of VOCs [38,40,41,42].

The main advantage of using NILs compared with other mapping populations is that QTLs can be located easily in defined introgressions flanked by molecular markers, where their effects can be studied in a homogeneous genomic background, normally from elite cultivars. Thus, NIL collections are considered efficient tools for studying the genetics of complex traits such as VOCs in agronomically important species, e.g., tomato [10,12,43] or melon [6,38,40,44].

The goal of the study was to map *ETHQB3.5* and examine its possible colocalization with other QTLs involved in the production of VOCs. This information will lead to classify VOCs as ethylene-dependent or independent. Putative candidate genes for climacteric ripening and VOC biosynthesis were tentatively located. Our null hypothesis is that the candidate gene involved in climacteric ripening boosts climacteric-dependent VOC biosynthesis.

## 2. Materials and Methods

### 2.1. Plant Materials, Crop Management, and Experimental Design

**Figure 1:**
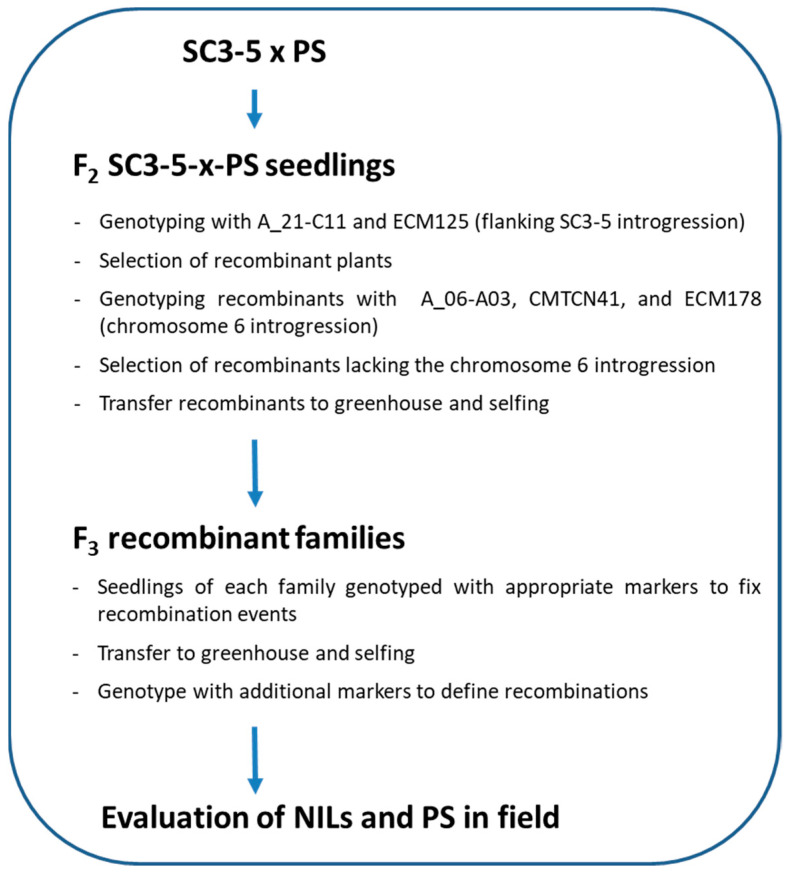
Mapping schedule used to obtain melon Near-Isogenic Lines (NILs) from SC3-5 and the parental PS.

A set of melon NILs with a single homozygous introgression in chromosome 3 from the Korean accession SC in the genetic background of the Spanish cultivar PS was selected for the present study. All the NILs were developed from the NIL SC3-5 that harbors an introgression in chromosome 3 from SC [37], as explained in the mapping schedule of Figure 1. Based on previous data [38,39], the location of *ETHQB3.5* was defined between markers ECM208 and ECM125 [45]. SC3-5 was backcrossed with the recurrent parent PS and F1 plants were selfed for subsequent recombinant screening. F2 seedlings were screened with the aforementioned markers, and plants showing recombinations among them were transferred to the greenhouse to be self-fertilized. Additional research showed that original NIL SC3-5 contained an introgression in chromosome 6, including the QTL *ETHQV6.3*, that is also involved in climacteric ripening **[32]**. In fact, SC3-5 showed SC alleles at markers ECM178, CMCTN41, and A06-C03 [45,46,47] (Table S1). Recombinant plants were genotyped with those markers, and those showing SC alleles were discarded. The recombination events were fixed in the next generation, and the extension of the recombinant introgressions was determined by genotyping with previously published mapped makers [24,25,46,47,48,49,50]. A total of six recombinant Introgression Lines (ILs) were obtained: SC3-5-7, SC3-5-8, SC3-5-12, SC3-5-13, and SC3-5-14) and were finally included in the experiments along with the recurrent parent PS.

Melon fruits used in this study were cultivated in an open field under Mediterranean conditions from April to July at the Integrated Center of Training and Agricultural Experimentation (CIFEA, in its Spanish acronym) located in Torre Pacheco (Murcia, Spain). The crop management techniques, harvesting practices, and harvest indices were those commonly used for this crop [39,40,51]. The numbers of replicates harvested (2–3 fruits per each of the replicate plants) were: *n* = 5 for SC3-5-7, *n* = 7 for SC3-5-8, and SC3-5-12 *n* = 9 for SC3-5-13 and SC3-5-14, and *n* = 21 for the parental PS used as reference. More replicates in PS were required to obtain better results using the Dunnett test after ANOVA. A completely randomized experimental design has been previously described [38,51,52].

### 2.2. Physiological Behavior and Maturity Indices at Harvest

The physiological behavior of the NILs and PS was confirmed during two seasons. According to [39,53], fruits were stored at 21 ± 1 °C and relative humidity of 66 ± 6% for at least 8 d. Respiration rate (CO_2_) and ethylene production (C_2_H_4_) were measured by the static method in at least 5 individual fruits of different replicate plants. Fruits were harvested from 7 to 10 a.m. at their optimum stage of maturity according to common harvest criteria [52]. Fruit density was measured at harvest by water displacement and fruit weight every day. The measurements were conducted at least 2 h after harvest to temperate and equilibrate the fruits with the environment. Fruits were enclosed in hermetic containers for 1 h and sampled for ethylene after 45 min and for CO_2_ after 1 h (or the equivalent time in order to avoid accumulation beyond 1% *w*/*w* CO_2_). The container was opened and the fruits were ventilated until the next-day measurement. Duplicate samples of 0.5 mL were removed every time from the headspace and analyzed via gas chromatography (GC) with Thermo Finnigan Trace GC 2000 (Milan, Italy) equipped with a thermal conductivity detector and 2-m Porapak N 80/100 column (Agilent J&W, EEUU, Santa Clara, CA, USA) for CO_2_ and a GS-Q de 30 m × 0.530 mm column (J&W Scientific, Agilent Technologies Inc., EEUU) and flame ionization detector for ethylene. 

GC conditions for CO_2_ were: oven temperature of 100 °C for 3 min and post-run conditions of 160 °C for 5 min with 70 kPa pressure. The injector and detector temperatures were 150 °C. A constant flow was used by mixing 30 mL He min^−1^ as inert gas and nitrogen as make-up gas of 10 mL min^−1^. The signal range was the maximum of 1.

For C_2_H_4_, oven temperature remained at 60 °C for 2.2 min, and then, a ramp of 80 °C·min^−1^ up to 80 °C was set. Every analysis lasted 2.45 min. The injector and detector temperatures were 200 °C and 250 °C, respectively. The split mode was 1:15 with a purge flow of 90 mL·min^−1^. The constant flow was established (6 mL·min^−1^) with a mixture of synthetic air (350 mL·min^−1^), H_2_ (35 mL·min^−1^), He (4 mL·min^−1^), and N_2_ (30 mL·min^−1^) as make-up gas. The signal range was 1.

The GCs were previously calibrated with external standards (1 ± 0.1 ppm for ethylene and 1 ± 0.1% *w*/*w* for CO_2_ -Air Liquide, Valencia, Spain-). Calculations were programmed in an Excel worksheet according to [54]. In accordance with their physiological behavior (levels of respiration rate and ethylene production), the NILs and PS (control) were classified as non-climacteric (NC), light, or moderately climacteric (LC or MC) [52]. The physiological behavior of the NILs was verified in two different seasons and *n* ≥ 5 fruits per season except for the NIL SC3-5-7 (only one season).

### 2.3. Juice Sampling and VOC Analysis

The methodology used to determine the VOC composition of melon juice by constant flow gas-chromatography mass-spectrometry (GC-MS) analysis was adapted from [38]. VOCs were tentatively identified and aligned by matching their mass spectra of individual components with those stored in the National Institute for Standards and Technology (NIST05a.L, search version 2.0) spectral database and by comparing with linear retention indices (LRI) reported in the literature or NIST database (http://webbook.nist.gov/chemistry/cas-ser.html (accessed on 1 January 2022)). VOCs were classified into ten classes of compounds (acetate esters, organic acids, alcohols, aldehydes, alkanes, ketones, non-acetate esters, sulfur-derived compounds, terpenes, and others). The variables of the compound classes and individual VOCs were obtained following a previously described method [44]. Consistently unidentified compounds (NID) of the untargeted GC-MS analysis were classified as unidentified and reported according to their mass spectra and/or LRI. The closest group of chemical compounds was tentatively reported from NID1 to NID4 compounds and used for the classification of compound classes. The NID1 compound with LRI 1143 was the same unidentified compound previously labeled as “NID3” [44].

### 2.4. Candidate Gene Selection

Molecular markers were anchored to the melon genome v4.0 [48,55] using the BLAST (Basic Local Alignment Search Tool) available at www.melonomics.net (accessed on 1 January 2022) (Table S1). Annotated genes included in the genomic regions defined by the markers were also retrieved from the Melonomics 4.0 website. The annotation of the genes was double-checked, also using BLAST to compare the annotated/predicted melon genes with the *Arabidopsis thaliana* database (TAIR; www.arabidopsis.org (accessed on 1 January 2022)) and the Uniref90 protein database (http://www.uniprot.org (accessed on 1 January 2022)). The annotation was retained when the significance of the BLAST comparison was *p* < 10^−5^. The role of transcription factors or other genes on the phenotype was not directly determined.

### 2.5. Statistical Analysis and QTL Mapping

The statistical analysis applied to the data from the compound classes and individual VOC variables has been explained previously in detail [44]. For each melon line, all individual VOCs and the classes of compounds were subjected to exploratory data analysis to detect possible outliers by using box-whisker plots allowing a visual comparison between NILs. The data were log-transformed (base 2) and, to apply this transformation, the zero values of VOCs were substituted by the minimum non-zero values observed in the whole dataset [12]. The transformed data were submitted to analysis of variance (ANOVA) with pedigree (NILs and PS) as a factor. The resulting *p*-values were corrected for multiple testing by using the Benjamini and Hochberg criterion [56]. Variables that showed significant differences at level *p* = 0.05 were selected for further analysis. NIL means were simultaneously compared with the control PS mean with a Dunnett’s test at level *p* = 0.05. NILs showing significant differences were assumed to harbor a QTL for the trait under study in their respective introgression from SC on chromosome 3. The position of the QTL was established by substitution mapping based on the different lengths of the introgressed segments [57]. 

A correlation network analysis (CNA) by considering the Pearson correlation coefficient was performed to evaluate the interactions among VOCs and to find groups containing highly correlated VOCs, which were visualized by drawing graphs. The pairs of compounds with absolute Pearson correlation coefficient values higher than a given threshold were selected. Additionally, a hierarchical cluster analysis (HCA) was carried out to classify and identify either cluster of VOCs or NILs. Specifically, an agglomerative hierarchical clustering (AHC) procedure was performed for the individual VOCs of PS and NILs together, considering the similarity metric defined in terms of the Pearson correlation coefficient and using the Ward’s minimum variance method. In general, AHC starts with the definition of each VOC belonging to a cluster. In each step, the closest pair of clusters are merged into a new cluster until all of the compounds are in a single cluster. In order to visualize the clustering procedure, a heatmap and a tree diagram (dendrogram) are represented by including the ordering of the clusters and the level of similarity or distance between clusters.

All statistical techniques were performed using free R environment software version 4.1.3 (R Foundation for Statistical Computing, Vienna, Austria) and the flashClust package for HCA and CNA [58]. The correlation networks and the heatmaps were visualized by using the igraph [59] and the gplot [60] packages, respectively.

### 2.6. Classification of VOCs According to Ethylene Dependence

The volatile compounds were classified as dependent or independent of ethylene action according to different criteria: (i) their levels in the NILs compared with PS and the physiological behavior of each NIL resulting from respiration and ethylene production measurements in separate fruits (climacteric or non-climacteric behavior) as reported above; and (ii) the colocalization of QTLs of VOCs and *ETHQB3.5*.

## 3. Results

### 3.1. Ideogram of the NILs. Physiological Behavior, Mapping of ETHQB3.5 and Putative Candidate Genes

**Figure 2:**
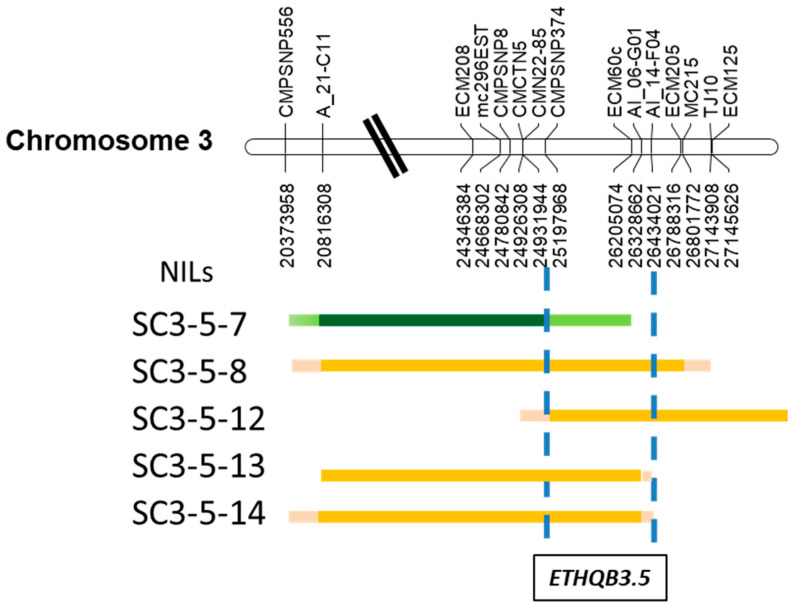
Ideogram showing the extent of the SC introgression on chromosome 3 among the Near-Isogenic Lines (NILs) and classification of their climacteric behavior (green—non-climacteric, orange—climacteric). Molecular markers are anchored to the melon genome v4.0. All the molecular markers are reported in Table S1. Pale or deep green color indicates introgressed region covered by non-climacteric NILs and pink–orange color by climacteric ones, according to Figure 3 and Figure S1. Pink and light green indicate the regions between markers where the recombination occurred in each NIL.

An ideogram showing the five NILs under study was obtained (Figure 2).

**Figure 3:**
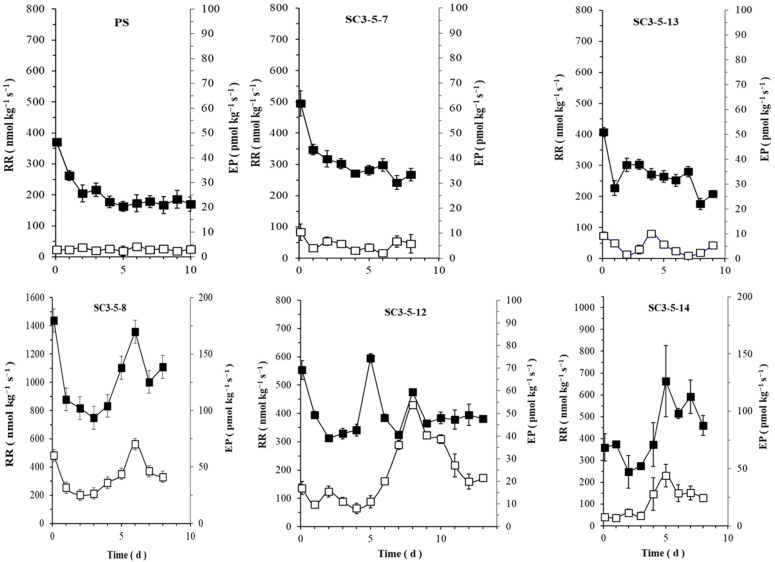
Respiration rate (CO_2_, ■) and ethylene production (C_2_H_4_, □) (RR and EP, respectively; mean ± S.E.) during postharvest ripening at 21 °C and relative humidity of 66 ± 6%. Non-climacteric fruits of parental PS (*n* = 21) and NIL SC3-5-7 (*n* = 5). Light–moderate climacteric fruits of NILs SC3-5-8, SC3-5-12 (*n* = 7), SC3-5-13 (*n* = 9), and SC3-5-14 (*n* = 9).

The parental PS and the NIL SC3-5-7 showed non-climacteric behavior, whereas three NILs (SC3-5-8, SC3-5-12, and SC3-5-14) were classified as climacteric. The NIL SC3-5-13 showed a climacteric respiration peak but was not accompanied by a consistent peak of ethylene production in the main season of the experiments (Figure 3). However, the climacteric pattern accompanied by the ethylene peak was confirmed during an additional season (Figure S1). The climacteric NILs showed moderate respiration rate levels of 250–1400 nmol∙kg^−1^∙s^−1^ of CO_2_ and a slight peak of ethylene production of 5–70 pmol∙kg^−1^∙s^−1^ of C_2_H_4_ (Figure 3).

In accordance with the above-mentioned classification of climacteric behavior (Figure 3), the QTL *ETHQB3.5* was mapped (Figure 2) between the molecular markers CMPSNP374 and AI_14-F04 of the melon genome v4.0 and separated by 1.24 Mb (physical positions from 25.197.968 to 26.434.021), e.g., less than 0.32% of the 400 Mb of the version 4.0 of the melon genome (Figure 2; Table S1, Table S2 and Table S3).

Because the role of transcription factor or genes on the phenotype was not directly determined, only a summary of the genes contained in the regions of potential interest in the genetic map were reported. One hundred and forty-seven genes were annotated within the genetic region that included *ETHQB3.5*, e.g., from MELO3C011093 to MELO3C011239, thirteen of them were with unknown functions that would require further characterization (Table S3). One hundred and forty-six additional genes were located in the introgression area studied outside of the *ETHQB3.5* region (forty-five genes from markers CMN22-85 and CMPSNP374; one hundred and one genes from markers AI_14-F04 and TJ10; Table S2 and Table S3). 

### 3.2. Volatile Organic Compounds

#### 3.2.1. Compound Classes of VOCs and Univariate Analysis

NILs and PS showed differences in compound classes and individual VOCs (Table 1 and Table 2). Aldehydes, alcohols, and ketones were the major VOC classes in PS and the NIL SC3-5-7. Ketones were significantly higher in the parental PS than in the NILs. Furthermore, higher aldehyde content was found in the NIL SC3-5-7 compared with PS. On the other hand, acetate esters and sulfur-derived compounds were more prominent among NILs except for SC3-5-7. The NIL SC3-5-8 showed relatively low levels of alkanes and terpenes compared with PS, whereas the level of compounds classified as “others” in all NILs (except for SC3-5-7) was lower than in PS (Table 1).

#### 3.2.2. Univariate Analysis of Individual VOCs

A total of 161 consistent volatiles were identified and subjected to univariate and multivariate analysis. One hundred and ten individual volatile compounds pointed out the main differences in aroma between PS and NIL (Table 2 and Table S4). They were mainly acetate esters, sulfur-derived compounds, and non-acetate esters, which showed values above those of PS or the non-climacteric NIL. Twenty-nine of the one hundred and ten compounds were absent in PS (mainly non-acetate esters and sulfur-derived compounds), and another thirty-five were present in one or several NILs irrespective of their physiological behavior (e.g., associated with this introgression in chromosome 3, Table 2). 

Hexanal was the most relevant in the percentage of total area (close to 8–20%), particularly above the upper level in parental PS and the non-climacteric NIL SC3-5-7. In PS, the content of the three volatile compounds, ethenylbenzene, (1-hydroxy-2,4,4-trimethyl-pentan-3-yl) 2-methylpropanoate, and 2-ethyl-3-hydroxyhexyl 2-methylpropanoate, was particularly high compared with the NIL.

Eight individual volatile compounds with higher levels in the climacteric NILs compared with PS (and one, ethenylbenzene, with a lower level in climacteric NILs than in PS) reflected the differences in physiological behavior between the climacteric NILs and PS. The eight individual compounds with higher relative levels in all the climacteric NILs were as follows: two acetate esters (benzyl acetate, butan-2-yl acetate), three non-acetate esters (methyl propanoate, 1-methylethyl acetate, 2-methylpropyl acetate), one sulfur-derived compound (1-(3-Hydroxypropylsulfanyl)ethanone), one alcohol (2-Methylbutan-1-ol), and one unidentified (NID1).

The difference in the aldehyde content between the non-climacteric NIL SC3-5-7 and PS was mainly due to NID4 and two aldehydes (2,4-dimethylbenzaldehyde; 4-methylbenzaldehyde) that were undetectable in PS. The other two aldehydes, (E)-hept-4-enal and (E)-non-2-enal, had a higher content in the NIL SC3-5-7 and other NILs (SC3-5-13 for (E)-hept-4-enal and SC3-5-14 for both compounds) than in PS (Table 2). On the other hand, the aldehydes (2E,6E)-nona-2,6-dienal and decanal were absent or present in lower concentrations, respectively, in NIL SC3-5-7 compared with PS (Table S5).

#### 3.2.3. Genes Located in the Region Mapped of ETHQB3.5 or Covered by the Introgression

The 1-deoxy-D-xylulose-5-phosphate synthase gene, a putative candidate gene for VOC biosynthesis, was mapped in the *ETHQB3.5* region (Table S3). The other two genes were found in the surrounding region of the SC introgression in melon chromosome 3, such as aspartate aminotransferase (MELO3C011284.2; data obtained from MELOGEN from physical positions 24913797 to 24917594), and a phospholipase (MELO3C011257) (Table S3). 

#### 3.2.4. VOC QTL Mapping

Several scenarios were obtained when we tried to map compound classes or individual volatiles (Table 3 and Table 4) by substitution mapping: (i) the inferred map position of the volatile QTL colocalized with *ETHQB3.5*, (ii) the QTL mapped in a different position than *ETHQB3.5* QTL, (iii) the segregation of the trait and introgressions among NILs was not compatible with the presence of a single QTL in the studied region. QTLs included in the (i) scenario are climacteric dependent and may be due to pleiotropic effects of *ETHQB3.5*, whereas QTLs included in the (ii) scenario would be genes linked to *ETHQB3.5* whose effects would be largely climacteric-independent. For example, QTLs for VOCs belonging to the acetate esters and sulfur-derived compounds classes colocalized with *ETHQB3.5*, whereas QTLs associated with aldehydes and ketones classes were mapped independently to *ETHQB3.5* (Table 1 and Table 3).

Forty-four VOC QTL could be mapped (only one per individual VOC) out of the one hundred and ten with some significant NIL effect in Table 2, and in thirty-one of which SC alleles increased volatile synthesis (Table 4). Twenty-one QTLs of individual VOCs colocalized with the region of *ETHQB3.5* (plus two more for compound classes of acetate esters and sulfur-derived compounds), and in nineteen of them (plus two of the compound classes mentioned above), the SC alleles increased the VOCs synthesis (Table 4 and Table S2). Most of them altered the composition of acetate and non-acetate esters and sulfur-derived compounds (Table 2, Table 4 and Table S2).

For VOCs without a QTL mapped in chromosome 3 (Table 2), a more complex genetic control was assumed. For example, for 25 VOCs, only SC3-5-8 showed significant differences compared to PS (Table 2). In another case, only SC3-5-7 showed an increased level of 2,4-dimethylbenzaldehyde or octyl acetate or a reduction in ethanol or decanal (Table 2), suggesting that *ETHQB3.5* or another tightly linked QTL present in the other NILs, modulates the effects of a QTL that is to the left part of the introgression that is covered by SC-3-5-7 (Table 4).

Twenty-five QTLs of VOCs were mapped in regions outside the region of *ETHQB3.5* in chromosome 3. Seventeen of the former QTLs (including aldehydes, ketones, and the rest of individual VOCs) were mapped to the right of the molecular marker AI_14-F04 (Table S2), and thirteen of them, including aldehydes, had a negative effect on the PS levels of VOCs. On the other hand, eight of the QTLs (seven VOCs plus ketones as compound classes) were mapped to the left of the molecular marker CMPSNP374 and showed a positive effect on VOC levels in PS, except for ketones as compound classes (Table 1, Table 2 and Table S2).

#### 3.2.5. Correlation Network and Hierarchical Clustering Analysis

**Figure 4:**
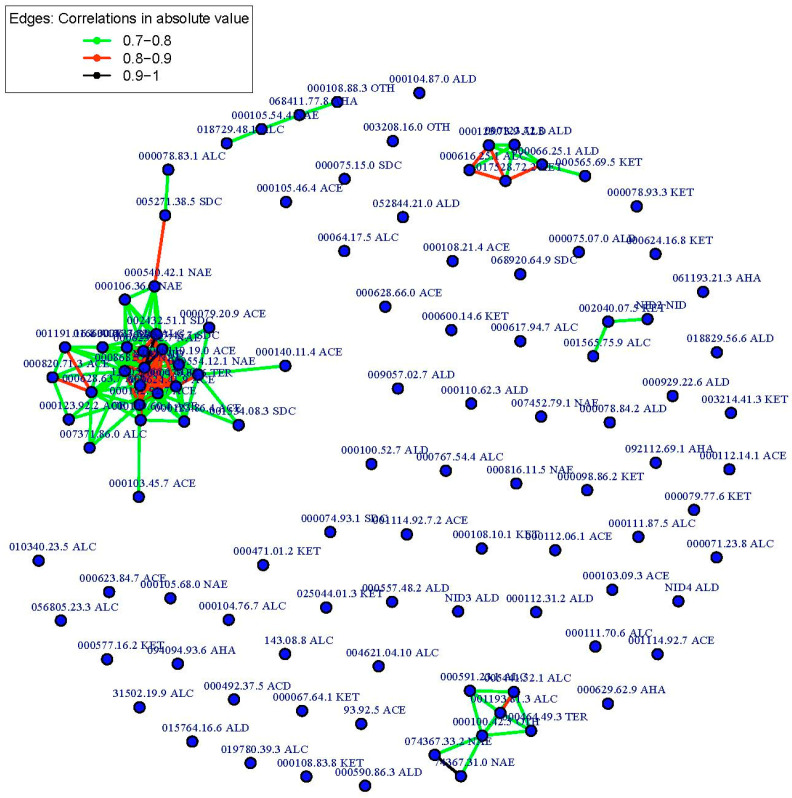
Correlation network analysis (CNA) based on absolute Pearson correlation coefficient with cut-off value greater than 0.7. Groups identified from individual volatile compounds analyzed for the Near-Isogenic Lines (NILs) with introgression on melon chromosome 3 (SC3-5-7, SC3-5-8, SC3-5-12, SC3-5-13, and SC3-5-14) and the parental control ‘Piel de Sapo’ (PS). CAS numbers use dots instead of dashes. ACE, acetate esters; NAE, non-acetate esters; SDC, sulfur-derived compounds; ALD, aldehydes; KET, ketones; ALC, alcohols; TER, terpenes; AHA, aliphatic compounds; OTH, other compounds; NID, unidentified compound.

**Figure 5:**
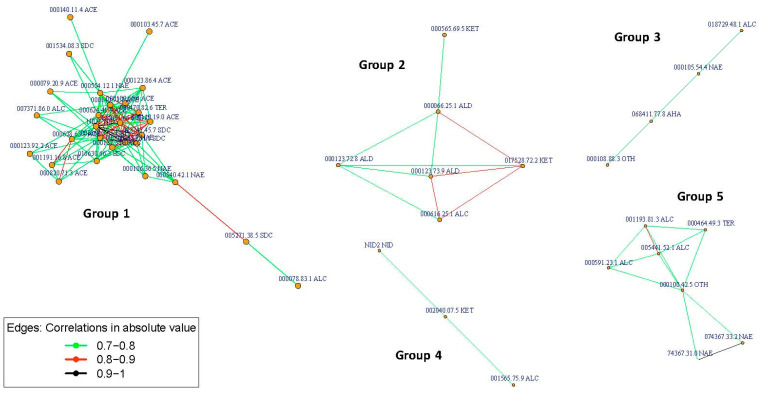
Grouping of individual volatile compounds with six or more compounds in each group obtained by correlation network analysis (CNA) applied to individual volatile compounds analyzed for Near-Isogenic Lines (NILs) with introgression on melon chromosome 3 (SC3-5-7, SC3-5-8, SC3-5-12, SC3-5-13, and SC3-5-14) and the parental control ‘Piel de Sapo’ (PS). CAS numbers use dots instead of dashes. ACE, acetate esters; NAE, non-acetate esters; SDC, sulfur-derived compounds; ALD, aldehydes; KET, ketones; ALC, alcohols; TER, terpenes; AHA, aliphatic compounds; OTH, other compounds; NID, unidentified compound.

The CNA built sixty-seven groups (Figure 4), five of which had at least three compounds (G1, G2, G3, G4, and G5) (Table 5), and the remaining only had one compound. In the most numerous group (G1), a network was established among twenty-eight volatile compounds (Table 2, Table 5 and Table S6; Figure 5); twenty-three of them were signed as important in the QTL identification in chromosome 3 (seventeen of them with QTLs colocalized with *ETHQB3.5*; Table 4, Table 5 and Table S6). Three sub-groups were obtained within G1, mainly associated with acetate esters, non-acetate esters, and sulfur-derived compounds, respectively. In G2, aldehyde, alcohol, and ketones were present. In the other groups, the grouping of VOCs was more difficult to classify, except in G5 (alcohol and non-acetate esters particularly, plus one terpene and one classified as another VOC type). In G2 and G5, most of the VOCs with QTL that mapped in chromosome 3 (four per group) were located to the right of *ETHQB3.5* and only for ethenylbenzene (in G5) colocalized with *ETHQB3.5*. The VOCs of G3 and G4 include compounds not interpretable in Table 4 for QTL mapping in chromosome 3 (Table 4, Table 5, Table S2 and Table S6).

**Figure 6:**
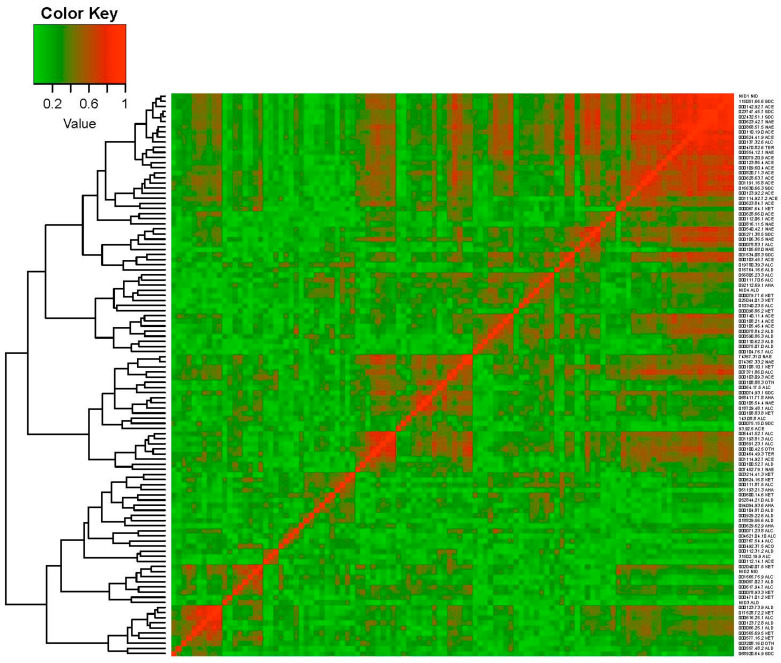
Heatmap of absolute Pearson correlation coefficients between pairs of individual volatile compounds was analyzed for the Near-Isogenic Lines (NILs) with introgression on melon chromosome 3 (SC3-5-7, SC3-5-8, SC3-5-12, SC3-5-13, and SC3-5-14) and the parental control ‘Piel de Sapo‘ (PS). Dendrogram obtained by Ward’s hierarchical clustering method was applied to the log_2_-transformed data. The individual volatiles are plotted by their respective CAS numbers (dots instead of dashes) on the right of the heatmap.

**Figure 7:**
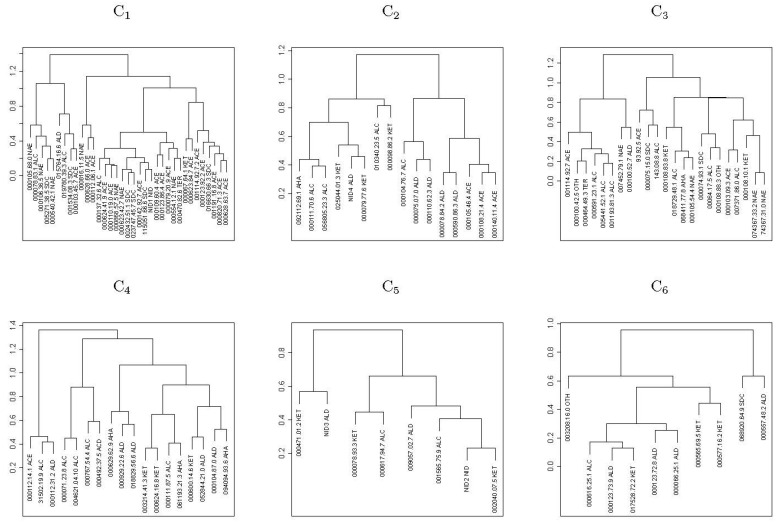
Detailed dendrograms of the six clusters (**C1**–**C6**) identified by hierarchical clustering applied to individual volatile compounds analyzed for the near-isogenic lines (NILs) with introgression on melon chromosome 3 (SC3-5-7, SC3-5-8, SC3-5-12, SC3-5-13, and SC3-5-14) and the parental control ‘Piel de Sapo’ (PS). The individual volatiles are plotted by their respective CAS numbers (dots instead of dashes) plus their chemical compound classes. ACE, acetate esters; NAE, non-acetate esters; SDC, sulfur derived compounds; ALD, aldehydes; KET, ketones; ALC, alcohols; TER, terpenes; AHA, aliphatic compounds; OTH, other compounds; NID, unidentified compound.

Six clustering VOCs can be clearly identified in the heatmap (Figure 6): C1, C2, C3, C4, C5, C6, and the detailed VOCs per cluster are shown in Figure 7. Twenty-six of the VOCs in C1 also belong to the most numerous group (G1) identified by correlation network analysis. As we mentioned above, these were important VOCs for QTL mapping and many colocalized with *ETHQB3.5* (Table 4). The VOCs included in the groups G3 and G5 were assigned to the same cluster, C3, while all VOCs in the groups G2 and G4 were identified into C6 and C5, respectively. Clusters C2 and C4 had no intercorrelated VOCs with higher correlation absolute values than 0.7.

**Figure 8:**
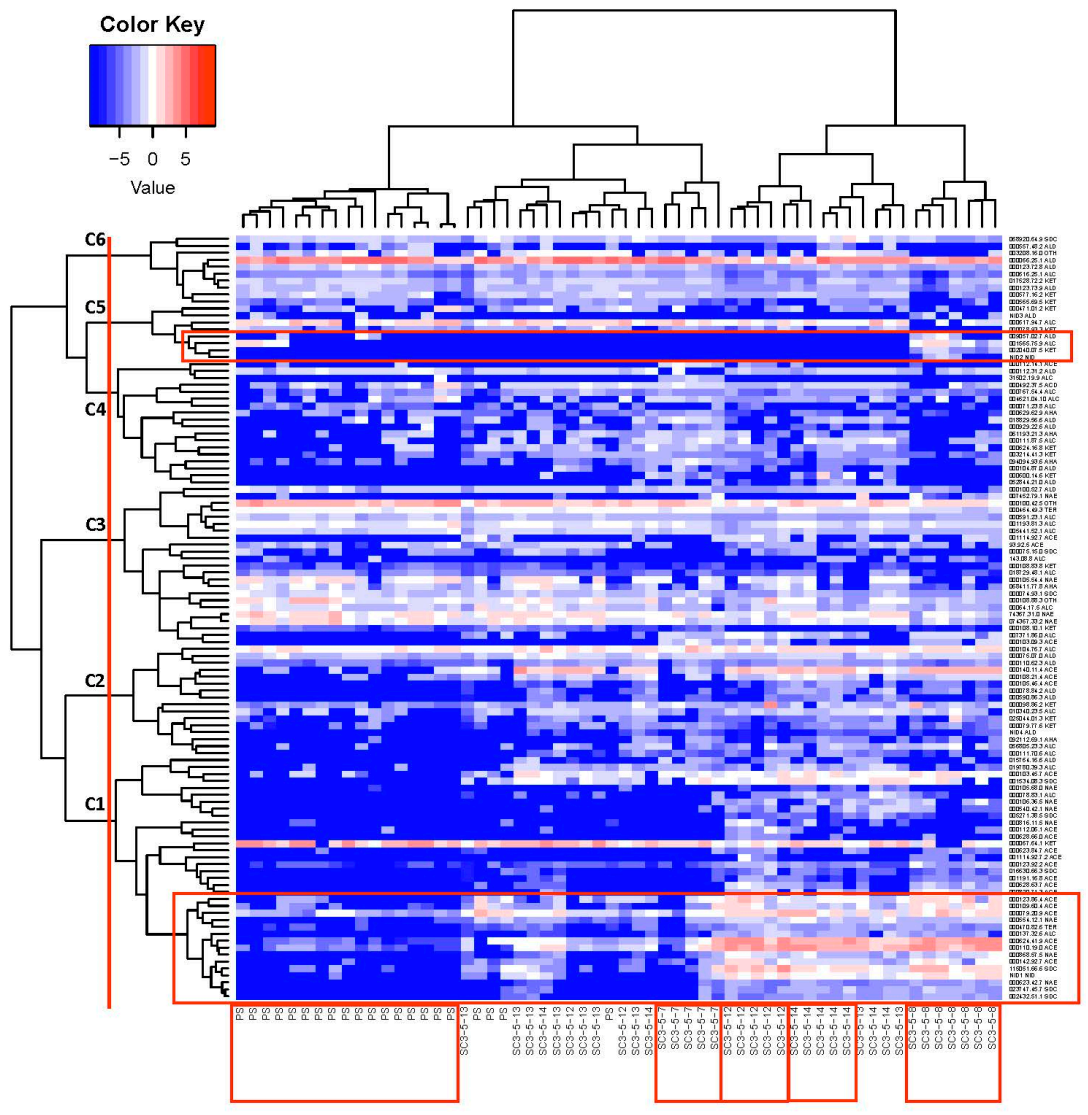
Heatmap for two-way hierarchical clustering applied to melon fruits and their aroma volatiles analyzed for the Near-Isogenic Lines (NILs) with introgression of melon chromosome 3 (SC3-5-7, SC3-5-8, SC3-5-12, SC3-5-13, and SC3-5-14) and the parental control ‘Piel de Sapo’ (PS). Dendrograms obtained by Ward’s hierarchical clustering method were applied to the log_2_-transformed data analyzed. The individual volatiles are plotted by their respective CAS numbers (dots instead of dashes) on the right of the heatmap.

A heatmap for two-way HCA of melon fruits and their VOCs indicates that cluster C1 showed more evident associations with climacteric NILs because the VOCs included in the cluster were related to esters (acetate and non-acetate) and sulfur-derived compounds (mostly esters) (Figure 8). These VOCs were produced via amino acids and, particularly, methionine. In fact, at the bottom left of the heatmap, the compounds that differentiate PS from the NILs are found, mostly esters, as expected. At this position, the separation between SC3-5-8, SC3-5-14, and SC3-5-12 from the rest was evident.

The VOCs in a sub-cluster C4 were mainly related to aldehydes (4-methylbenzaldehyde; 1-cyclohexene-1-carboxaldehyde, 2,6,6-trimethyl-; (E)-hept-4-enal) and ketones (pentane-2.3-dione; octane-2-5-dione) and were associated to the non-climacteric NIL SC3-5-7, while the VOCs included in cluster C5 (top of Figure 8, VOCs located on the right), such as propanal, 2-phenylbutan-2-ol, 1-(2,4,5-trimethylphenyl)ethanone or butan-2-one, were associated with the climacteric NIL SC3-5-8, which showed an exclusive different level of these VOCs versus PS (Table 2). Consequently, these VOCs were not interpretable for QTL mapping (Table 2 and Table 4).

### 3.3. Association between VOCs and Climacteric Behavior

The QTL of twenty-one individual VOCs mapped in the *ETHQB3.5* region exclusively showed ethylene dependence (plus the acetate esters and sulfur-derived compounds as compound classes). Twenty-three QTLs (plus the aldehydes and ketones as compounds classes) showed full ethylene independence (Table 3, Table 4 and Table S2).

## 4. Discussion

The melon aroma was complex and strongly dependent on the NIL or cultivar studied, but also on physiological behavior (Figure 3), in agreement with previous results [6,20,38,40,43,62]. Many studies comparing climacteric and non-climacteric cultivars of different origins reached a similar conclusion concerning the predominance of aldehydes and ketones in non-climacteric types and different levels of esters in climacteric ones [62,63,64,65,66], all in a ripening-dependent manner [18,36,40,67]. However, the present study details that the components of climacteric melon aroma, such as acetate esters and sulfur-derived compound classes (and several individual VOCs) colocalized with *ETHQB3.5* (Table 3 and Table 4). The former results are supporting the link between this QTL and QTLs of individual VOC production.

In detail, climacteric NILs and PS showed different accumulation levels of certain individual VOCs, mainly acetate esters (e.g., 1-methylethyl acetate or 2-methylpropyl acetate) and sulfur-derived compounds (e.g., 1-methylsulfanylbutan-1-one or 3-methyl-1-methylsulfanyl-butan-1-one) (Table 2). Three of these acetate esters (1-methylethyl acetate, 2-methylpropyl acetate, and benzyl acetate), which are related to the climacteric behavior of NILs and their QTLs colocalized with *ETHQB3.5* (Table 4), and three acetate esters identified from our results (hexyl acetate, propyl acetate, and methyl acetate) (Table 2) have been previously reported in NILs with introgression in chromosomes 3 and/or 6 [38,40]. In general, as in [38,40], all these compounds, except 1-methylethyl acetate with its lower values, showed higher values in the NILs than in PS.

The importance of ethylene-dependent esters (particularly acetate esters) and sulfur-derived compounds (collectively or individually) has also been outlined in the flesh of intact fruits of the climacteric NIL SC3-5-1 containing both introgression in melon chromosomes 3 and 6 (and the corresponding QTLs *ETHQB3.5* and *ETHQV6.3*), which indicated that our results using melon flesh in these NILs are not an artifact [20,36,42]. In addition, thirteen of the compounds reported in intact fruit are described as important in chromosome 3 in the present investigation with VOCs obtained from pulp juice, but not all of them have QTLs that colocalized with *ETHQB3.5* (Table 2, Table 4 and Table S2). Among them, the QTLs of 2-methylbutyl acetate, hexyl acetate, 2-phenylethyl acetate, or S-methyl ethanethioate did not colocalize with *ETHQB3.5*, while butyl acetate and 3-methyl-1-methylsulfanyl-butan-1-one colocalized with *ETHQB3.5* (Table 2, Table 4 and Table S2). This result is an indication of potential ethylene dependence and independence of VOCs of interest in melons.

Several of the esters (acetate and non-acetate) identified (Table 2 and Table S4) have been described as predominant in climacteric orange-flesh cantaloupes (*C. melo* var. reticulatus, Naudin, cv. Sol Real) but are also important in oriental melons. In cantaloupes, most of them were acetate ester-type: methylbutyl (e.g., 2-methylbutyl acetate, 3-methylbutyl acetate), ethyl (e.g., ethyl butanoate), hexyl (e.g., hexyl acetate), nonenyl and benzyl (e.g., benzyl acetate), and non-acetate ester-type: butanoates (e.g., ethyl butanoate), methylpropanotaes, and hexanoates. Alcohols (e.g., 2-methylbutan-1-ol) and aldehydes were described as important precursors in the synthesis of key esters of melon aroma [67,68,69,70]. In climacteric oriental Hami-type melon, acetate esters (e.g., ester 2-methylpropyl acetate) and non-acetate esters (e.g., methyl 2-methylbutanoate) have also been reported [71]. The former authors reported a profile with many other VOCs similar to those in the climacteric NILs under study (Table 2 and Table S4), probably due to the origin of the introgression of the Korean accession PI161375 [37].

Essentially, *ETHQB3.5* in the PS genetic background (Figure 2) enhanced overall ester VOCs, including acetate and sulfur-derived esters and some non-acetate esters. Santo Domingo et al. [36] suggested that *ETHQB3.5* in PS background does not produce a strong effect in the main contributors to the melon aroma, at least compared with the other genetic backgrounds, such as ‘Védrantais’, and that the introduction of other climacteric QTLs is necessary for designing a strong aromatic melon line. However, the study of *ETHQB3.5* is enough to study VOC’s ethylene dependence or independence. We compared our results with VOCs obtained via hybrids of/and ACO (antisense aminocyclopropane-1-carboxylic acid oxidase) in ‘Védrantais’ [72,73] or antisense suppression of alcohol acetyltransferase in Hami melons [74]. In general, the agreement with the enhancement of many ester VOCs and those of the antisense ACO could be due to the concomitant increase of many volatiles of the same pathway and the coordinated expression of the genes associated with this pathway (Table 4). Even so, some discrepancies are apparent when comparing some ester VOCs classified as ethylene-independent (Table 2 and Table 4) and the apparent full ethylene-dependence of esters apparently synthesized from alcohols by alcohol acetyl transferases (*Cm*AAT) [73]. The potential explanation for this apparent discrepancy (for example, for 2-methylbutyl acetate, a VOC with QTL classified as ethylene-independent, Table 4 and Table S2) is the presence of alternative pathways for ester biosynthesis in melon (yet to be confirmed), similar to how it happens in apple [75].

On the other hand, the antisense Hami oriental melons that affected the last step of conversion of alcohols into esters [74], reduced the level of many esters (2-methylpropyl acetate, butyl acetate, 1-methylsulfanylbutan-1-one, 3-methylbutyl acetate, hexyl acetate, benzyl acetate, 2-phenylethyl acetate, and methyl acetate). They were characteristic compounds in the climacteric NILs with high levels in general, but not in all of them their QTLs were mapped within the *ETHQB3.5* region (Table 4).

One possible hypothesis for discrepancies with the former authors is the differences due to the genetic background [36], or that the alteration of climacteric ripening by *ETHQB3.5* had pleiotropic effects on the ethylene and VOC biosynthesis that could be controlled by genes widely distributed across the melon genome [36], as it has been revealed by the presence of potential *cis* e-QTLs [76].

In climacteric NILs, the higher content of individual non-acetate esters (e.g., propyl propanoate and methyl butanoate) absent from PS and absent in non-climacteric NILs suggests again a pleiotropic effect and only partial dependence on ethylene of this compound class, as has been outlined for other quality traits with the QTLs of chromosome 3 [32,52]. The non-acetate esters methyl propanoate and methyl butanoate, have previously been reported in climacteric NILs with higher values than in PS [38,40], and both were classified as ethylene-dependent here (Table 4 and Table S2). The non-acetate esters methyl 2-methylbutanoate and ethyl butanoate have also been previously reported by [38,40] where they showed higher values in NILs (climacteric and non-climacteric) than in PS. In fact, this could be the reason for ethylene-independence or lack of classification, respectively, in our case (Table 2, Table 4 and Table S2)

Although the climacteric trait in our NILs acts as a powerful enhancer in the production of many VOCs (Table 2), some of them also increased in non-climacteric NILs compared to parental PS. This suggests an additional explanation, which is that at least two QTLs could exist for this compound, the main one, ethylene-dependent, and the minor one, ethylene-independent. Unfortunately, we were unable to map two QTLs with this pattern, though this might be the case of a lack of QTL interpretation associated with the production of certain VOCs, particularly esters (Table 2). We hypothesize that this might be due to an additional QTL in other non-target introgressions that may also segregate in the genetic background.

The production of aldehydes, ketones, and alcohols was partly ethylene-independent because the levels of the individual volatile compounds belonging to these classes of compounds (Table 1 and Table 2) were directly related to the separation according to climacteric or non-climacteric behavior of the NILs, in agreement with many authors [65,66,77]. One relevant case was 2-methylbutan-1-ol with relative levels higher in climacteric NILs, a reason why its QTL mapped within the *ETHQB3.5* region (Table 2 and Table 4). In this sense, the introgression in non-climacteric NIL SC3-5-7 induced an increase in the aldehyde content compared with PS aroma, particularly in several individual compounds, as also observed in HCA figures (Table 2, Tables S4 and Table S5; Figure 7 and Figure 8). Thus, this introgression, without carrying any QTL or gene that alters the climacteric behavior, had the potential to change the aroma of PS, as has been detected in NILs with introgression in chromosome 3 or other chromosomes [6,38,44]. Furthermore, the NIL SC3-5-7 showed reduced terpene content (Table 1), which might be affected by 1-deoxy-D-xylulose-5-phosphate synthase [15], a gene that maps in the region of recombination of this NIL located between the molecular markers CMPSNP374 and ECM60c (Table 4 and Table S3).

The coexistence of the dependence and independence of VOC production on ethylene suggested that our results agreed with [78] and are partly due to the different pathways to produce branched-chain ester or straight-chain esters, also depending on their precursors. According to [67], the metabolism of branched-chain amino acids, including Val, Leu, Ile, as well as the Phe as an aromatic amino acid, and Cys as a sulfur-containing amino acid, are under the regulation of ethylene. When Flores et al. [78] studied the formation of hexyl acetate from hexanal or butyl acetate from butanal, they concluded that the reduction of fatty acids and aldehydes was essentially an ethylene-dependent process, while the following process of ester-formation included ethylene-independent components too. In fact, we located a QTL ethylene-independent for hexanal, hexyl acetate, or butanal, but for butyl acetate, the QTL mapped was fully ethylene-dependent (Table 4 and Table S2). Factors, other than their physiological behavior but associated with the genotype of each NIL (such as internal characteristics of the different NILs) and conditioned by the introgression, could be critical in the production of these VOCs.

The use of mapping in this experiment found clustering of QTLs that apparently controlled the levels of VOCs, belonging to similar compound classes linked to the genomic region that contain the climacteric QTL *ETHQB3.5* (Figure 4, Figure 5, Figure 6, Figure 7 and Figure 8). Linkage of *ETHQB3.5* to other VOC QTLs in the original SC3-5 introgression could be the reason for the ethylene independence of some VOCs found in this work that have been previously reported as ethylene-dependent [36]. Additionally, the correlations of VOCs in HCA or CNA (Figure 4, Figure 5, Figure 6, Figure 7 and Figure 8) are consistent with the existence of a few genomic regions controlling the levels of most of the investigated VOCs and with the clustering of QTLs for the same compound class, in agreement with results in peach [5]. Clustering of QTLs controlling VOCs with similar chemical structures has been found in apples [2], tomatoes [79], strawberries [16], and peaches [5], but this pattern could also be associated with pleiotropic effects of a single locus or a tight linkage between different loci. Though this genetic variation in climacteric pattern and VOC production could be important and has significant agricultural value by using DNA-based molecular markers in plant breeding programs, new findings on epigenetic variation controlling fruit development and ripening should be considered for future studies [80,81].

The potential precursors of the VOCs, depending on their chemical groups, are relevant for the discussion of the HCA (Figure 6 and Figure 7; Table 5 and Table S6). Some amino acids (Val, Phe, Ile, Leu, Thr, Ala and Met) are the main precursors involved in metabolic pathways of some of the aroma volatiles according to the literature (Tables S4 and Table S6). The amino acids Val, Ile, Met, and Ala have been postulated as the precursors of most of the esters found in our melon fruits (Table 2, Tables S4 and Table S6; [67]). The amino acids Val, Ile, and Met were the main precursors putatively responsible for the production of the volatiles that colocalized with *ETHQB3.5* (Table S4). More specifically, the data and references of Table S4 suggest that the metabolism of amino acids (mainly Met in sulfur-derived compounds, Ile in non-acetate esters and Ala, and Phe in acetate esters) is associated with volatile production. Treatment with 1-methylcyclopropene (1-MCP), a competitor of ethylene for binding to the ethylene receptor, affects the biosynthesis of flavor volatiles, such as acetate esters derived from the amino acids Ile and Phe, in cut climacteric Cantaloupe melons stored at 5 °C, but also reduced the amount of sulfur-derived compounds [82]. In general, our results (Table 1) are consistent with those former findings, but in our study, the effect of 1-MCP was not significant with some individual VOCs tightly linked to *ETHQB3.5* (e.g., for butyl acetate or 2-methylpropyl acetate), or even its effect was the opposite (e.g., for 2-methylbutyl acetate).

On the other hand, linolenic and linoleic fatty acids have also been described as the main substrates involved in the synthesis of acetate and non-acetate esters [63,83,84]. The amino acids (mainly Ala) and linolenic acid have been reported by the former authors (Table S6) to act as precursors of acetate esters (first subcluster in HCA). Three important compounds were located in this subcluster. The 2-methylbutyl acetate is an odorant with an intermediate intensity, very abundant in climacteric Cantaloupe-type, Charentais-type, and oriental Jiashi-type melons [68,71,77], and is predominant, together with butyl acetate and hexyl acetate, in Galia-type melons, both in the whole fruit and in the flesh [42,77,85]. According to the literature, the precursors of 2-methylbutyl acetate are the amino acids L-Ala and L-Ile, whereas linolenic acid is the precursor of hexyl acetate (CAS 142-92-7) and butyl acetate (CAS 123-86-4) (Table S4).

Among the above CNA subclusters (Figure 8), sulfur-derived compounds are probably more important because of their potential involvement in the regulation of Yang’s cycle [86], which uses L-Met as a precursor. Sulfur-derived compounds are important volatiles in melon aroma, L-Met degradation via methionine-γ-lyase, and the catabolic route through methionine aminotransferase being key features in the formation of such compounds and other melon volatiles [63,84,87]. One of these sulfur-derived compounds, methyl 2-methylsulfanylacetate, was present at very high levels in our climacteric NILs (Table 2 and Table S4) and in other climacteric cultivars [64]. The important presence of sulfur-derived ester volatiles in climacteric NILs suggests the possible regulation of ethylene production in climacteric NILs through the conversion of excess L-Met into sulfur-derived volatiles by this degradation pathway. A similar hypothesis regarding the regulation of some esters by precursor availability has been suggested in *Actinidia chinensis* [88]. Some sulfur-derived compounds have been found in intact climacteric NILs containing the QTLs *ETHQB3.5* and *ETHQV6.3*, such as the SC3-5-1 [42], and are, therefore, not artifacts associated with the extraction and analysis procedures reported in other systems [89].

Among the candidate genes located in the *ETHQ3.5* QTL region, MELO3C011118, involved in the ethylene-activated signaling pathway [90] and stress response (Table S3), could also be directly involved in ethylene production associated with the climacteric pattern because, according to UNIPROT database (http://www.uniprot.org/uniprot/Q8RY59 (accessed on 1 January 2022)), the gene also interacts with dehydration-responsive DREB2 proteins and several transcription factors belonging to several protein families.

On the other hand, MELO3C011229 codifies an EG45-like domain-containing protein involved in alternative respiration (Table S3) and can be of interest because the cyanide-insensitive respiration contributes to the rise in respiration rates reported in several fruits in climacteric and ethylene-mediated ripening [91,92]. Another gene (MELO3C011217) codifies the Protein TIFY 8 involved in the jasmonic-acid-mediated signaling pathway in *Arabidopsis thaliana* (Table S3). Furthermore, MELO3C011093 is a MADS-box protein, and MELO3C011103 is a MADS-box interactor-like, and these genes play a major role in fruit development and ripening [93]. MELO3C011118 is associated with stress response, particularly the ethylene-activated signaling pathway (Table S3). Finally, there were other genes in the *ETHQB3.5* region (Table S3) that could be of interest. MELO3C011164 is a NAC domain-containing protein 92, and, interestingly, the gene underlying the climacteric QTL *ETHQV6.3* encodes the NAC transcription factor *Cm*NAC-NOR [32,94]. Therefore, this NAC transcription factor should be taken into consideration to be included in the NAC positive feedback loop that operates in climacteric melons [95,96].

## 5. Conclusions

*ETHQB3.5* has been mapped in a 1.24 Mb genome region, setting the basis for its future molecular cloning. The accumulation of VOCs, mostly from the acetate esters, non-acetate esters, and sulfur-derived compound families, most likely has been associated with pleiotropic effects of the alteration of climacteric ripening by *ETHQB3.5* rather than structural genes located in this region of melon chromosome 3.

Some QTLs for the VOCs (twenty-three in total) colocalized with *ETHQB3.5* (e.g., 3-methylbutyl acetate, benzyl acetate, 2-methylbutan-1-ol, 2-methylpropyl acetate, and 1-methylsulfanylbutan-1-one), while others did not map on chromosome 3 (ethyl butanoate and methyl acetate) or mapped nearby (2-methylbutyl acetate, hexyl acetate, and 2-phenylethyl acetate). Therefore, the ethylene dependence of many VOCs must be considered in future breeding programs.

VOCs from the ketones and aldehyde compound families showed an accumulation independent of *ETHQB3.5*, indicating that genes involved in the content of these VOCs would be present in other regions of the same chromosome 3. Taken together, the results show that the modification of melon aroma may be achieved with or without interfering with its physiological behavior. The production of high levels of some ethylene-dependent esters and sulfur-derived compounds identified here would require the active participation of the climacteric QTL *ETHQB3.5*.

## Figures and Tables

**Table 1:** Main compound classes identified for QTL mapping in melon chromosome 3 using five Near-Isogenic Lines (NILs) with different physiological behavior. Data are expressed as percentages of each compound class with respect to the total normalized areas.

NILs. Replicates and Classification of Climacteric Behavior ^a^													
	PS	SC3-5-7		SC3-5-8		SC3-5-12		SC3-5-13		SC3-5-14			*ETHQB3.5* Association ^d^
	*n* = 21	*n* = 5		*n* = 7		*n* = 7		*n* = 9		*n* = 9			
Compound Class ^b^	(NC)	(NC)		(LC/MC)		(LC/MC)		(LC/MC)		(LC/MC)		*p*-Value ^c^	
ACE	5.89	7.24		43.24	*	46.39	*	15.73	*	24.56	*	****	Full
NAE	6.31	5.38		4.90		5.47		6.36		6.00		NS	No
ALD	33.30	52.02	*	19.24		13.64	*	35.82		29.44		***	No
ALC	16.80	17.17		14.78		11.40		15.54		16.05		NS	No
KET	19.80	9.48	*	6.79	*	6.35	*	13.00	*	10.06	*	****	No
ACD	0.24	0.38		0.36		0.30		0.23		1.02		NS	No
SDC	1.50	2.22		7.79	*	7.25	*	4.84	*	6.99	*	****	Full
TER	1.40	1.01		0.73	*	0.97		1.36		1.31		***	No
AHA	2.08	1.69		0.58	*	1.20		1.96		1.27		**	No
OTH	6.42	3.51		1.63	*	2.62	*	4.36		3.36	*	****	No

^a^ Non-climacteric, NC; LC or MC Light or moderate climacteric, respectively. ^b^ Compound classes: ACE, acetate esters; NAE, non-acetate esters; ALD, aldehydes; ALC, alcohols; KET, ketones; ACD, organic acids; SDC, sulfur-derived compounds; TER, terpenes; AHA, alkanes; OTH, others. Underlined terms indicate absence of QTL mapped in melon chromosome 3 due to the lack of interpretation of the results. ^c^ NIL means followed by an asterisk were significantly different from the control PS according to one-way ANOVA with pedigree effect followed by a Dunnett test (*p* = 0.05). *, **, ***, ****: significance at *p* ≤ 0.05, 0.01, 0.001, and 0.0001, respectively. NS, non-significant. ^d^ Association of the group of aroma volatiles with the presence of *ETHQB3.5* in the map (Figure 3) without taking into account SC3-5-13.

**Table 2:** Main fruit volatile compounds identified for QTL mapping in melon chromosome 3 using five Near-Isogenic Lines (NILs) with different physiological behavior (SC3-5-7, *n* = 5; SC3-5-8 and SC3-5-12, *n* = 7; SC3-5-13 and SC3-5-14, *n* = 9) and the parental control ‘Piel de Sapo’ (PS) (*n* = 21). Volatile compounds were selected for significance of the univariate statistical criterion described. Data are the mean relative content in percentage with respect to the total of the probable aromatic volatile compounds identified. NIL means in bold followed by an asterisk were significantly different from the control PS mean according to one-way ANOVA with pedigree effect followed by a Dunnett test (*p* = 0.05). *, **, ***, **** significance at *p* ≤ 0.05, 0.01, 0.001, and 0.0001, respectively.

					NILs and Classification of Climacteric Behavior ^a^												
			Compound		PS	SC3-5-7		SC3-5-8		SC3-5-12		SC3-5-13		SC3-5-14			
Order ^b^	CAS ^c^ Number	IUPAC ^d^ Name	Class ^e^	IDN ^f^	(NC)	(NC)		(LC/MC)		(LC/MC)		(LC/MC)		(LC/MC)		*p*-Value ^g^	MQ ^h^
1	000554-12-1	Methyl propanoate	NAE	17	0.06	0.13		**0.3**	*	**0.24**	*	**0.12**	*	**0.45**	*	****	95
2	000108-21-4	1-Methylethyl acetate	ACE	21	0.05	0.15		**0.36**	*	**0.37**	*	**0.31**	*	**0.47**	*	****	81
3	001534-08-3	S-Methyl ethanethioate	SDC	27	0.05	**0.82**	*	**0.92**	*	**0.74**	*	**0.98**	*	**1.44**	*	****	87
4	000109-60-4	Propyl acetate	ACE	32	0.16	0.41		**1.61**	*	0.85		0.25		0.21		**	83
5	000623-42-7	Methyl butanoate	NAE	33	0	0.02		**0.11**	*	**0.07**	*	0.02		**0.08**	*	****	91
6	000137-32-6	2-Methylbutan-1-ol	ALC	34	0	0.03		**0.09**	*	**0.15**	*	**0.04**	*	**0.16**	*	****	86
7	000110-19-0	2-Methylpropyl acetate	ACE	44	0.06	1.79		**8.33**	*	**6.17**	*	**2.39**	*	**5.45**	*	****	83
8	000868-57-5	Methyl 2-methylbutanoate	NAE	45	0	**0.07**	*	**0.46**	*	**0.42**	*	**0.06**	*	**0.24**	*	****	76
9	000105-54-4	Ethyl butanoate	NAE	49	0.18	0.82		**0.54**	*	0.85		0.39		0.13		****	97
10	000106-36-5	Propyl propanoate	NAE	50	0	0.03		**0.32**	*	**0.11**	*	0.09		**0.11**	*	****	78
11	000123-86-4	Butyl acetate	ACE	51	0.17	0.41		**2.31**	*	**1.99**	*	0.39		**0.46**	*	***	90
12	002432-51-1	1-Methylsulfanylbutan-1-one	SDC	55	0	0.05		**0.12**	*	**0.06**	*	0.04		**0.1**	*	****	63
13	000123-92-2	3-Methylbutyl acetate	ACE	64	0.02	0.03		**0.27**	*	**0.16**	*	0.04		**0.05**	*	****	90
14	000624-41-9	2-Methylbutyl acetate	ACE	65	0.13	**1.19**	*	**10.04**	*	**9.16**	*	**1.24**	*	**4.18**	*	****	83
15	000100-42-5	Ethenylbenzene	OTH	66	5.21	3.14		**2.17**	*	**2.37**	*	**3.12**	*	**3.24**	*	***	97
16	016630-66-3	Methyl 2-methylsulfanylacetate	SDC	72	0	0.01		**0.05**	*	**0.09**	*	0.01		**0.03**	*	****	87
17	001191-16-8	3-Methylbut-2-enyl acetate	ACE	73	0	0.01		**0.06**	*	**0.06**	*	0.01		0.01		****	72
18	023747-45-7	3-Methyl-1-methylsulfanyl-butan-1-one	SDC	74	0	0.04		**0.18**	*	**0.08**	*	0.04		**0.13**	*	****	64
19	000111-70-6	Heptan-1-ol	ALC	83	0	**0.03**	*	0.01		**0.03**	*	**0.05**	*	**0.06**	*	***	80
20	115051-66-6	1-(3-Hydroxypropylsulfanyl)ethanone	SDC	94	0.07	0.72		**3.82**	*	**4**	*	**0.59**	*	**1.16**	*	****	64
21	000142-92-7	Hexyl acetate	ACE	96	0.02	**0.1**	*	**0.81**	*	**1.2**	*	**0.11**	*	**0.25**	*	****	85
22	000470-82-6	1,8,8-Trimethyl-7-oxabicyclo[2-2-2]octane	TER	98	0.01	0.04		**0.11**	*	**0.13**	*	0.04		**0.1**	*	****	96
23	019780-39-3	(2R,3S)-3-Ethylheptan-2-ol	ALC	106	0.03	**0.14**	*	0.03		**0.2**	*	**0.16**	*	**0.25**	*	****	50
24	NID1	NID1 (LRI 1143; RT 20.871) (m/z 43, 88, 73, 61, 148, 41, 45)	NID	122	0.02	0.15		**1.67**	*	**1.58**	*	**0.27**	*	**0.8**	*	****	55
25	3901-95-9	1-Methyl-4-propan-2-ylcyclohexan-1-ol	ALC	125	0.1	**0.21**	*	0.06		0.11		**0.17**	*	**0.29**	*	****	71
26	000140-11-4	Benzyl acetate	ACE	132	0.18	0.16		**5.08**	*	**2.97**	*	**3.21**	*	**3.34**	*	****	97
27	056805-23-3	(3Z,6Z)-Nona-3.6-dien-1-ol	ALC	137	0.02	**0.15**	*	**0.3**	*	**0.26**	*	0.12		0.23		***	80
28	007371-86-0	4-Acetyloxypentan-2-yl acetate	ALC	141	0.02	**0.28**	*	**0.43**	*	**0.59**	*	0.06		**0.18**	*	****	73
29	015764-16-6	2,4-Dimethylbenzaldehyde	ALD	149	0	**0.13**	*	0.03		0.06		0.1		0.04		**	93
30	000103-45-7	2-Phenylethyl acetate	ACE	151	0.12	**0.77**	*	**1.73**	*	**1.07**	*	**0.84**	*	**1.01**	*	****	72
31	074367-33-2	(1-Hydroxy-2.4.4-trimethyl-pentan-3-yl) 2-methylpropanoate	NAE	153	1.42	0.55		**0.34**	*	**0.45**	*	1.02		0.87		***	78
32	000067-64-1	Acetone	KET	4	6.26	2.62		**1.06**	*	4.96		4.96		3.99		**	72
33	000079-20-9	Methyl acetate	ACE	6	0.69	0.58		**2.25**	*	1.13		1.13		**1.77**	*	*	86
34	000078-84-2	2-Methylpropanal	ALD	7	0	0.02		**0.06**	*	0.02		0.02		**0.17**	*	****	81
35	000078-83-1	2-Methylpropan-1-ol	ALC	16	0	0.04		0.01		**0.04**	*	**0.04**	*	**0.27**	*	**	68
36	000108-88-3	Methylbenzene	OTH	41	1.59	**0.33**	*	**0.25**	*	1.4		0.87		0.93		**	76
37	003214-41-3	Octane-2-5-dione	KET	87	0.03	**0.1**	*	0.04		0.09		**0.09**	*	**0.08**	*	**	71
38	000111-87-5	Octan-1-ol	ALC	110	0.05	**0.51**	*	0.1		**0.21**	*	0.18		**0.51**	*	****	72
39	000079-77-6	(E)-4-(2,6,6-Trimethyl-1-cyclohexenyl)but-3-en-2-one	KET	160	0.02	**0.11**	*	**0.06**	*	0.02		**0.07**	*	**0.09**	*	****	71
40	000075-07-0	Acetaldehyde	ALD	1	0.25	0.38		0.36		0.28		**0.48**	*	**0.48**	*	*	83
41	000074-93-1	Methanethiol	SDC	2	0.35	0.21		**0.16**	*	**0.17**	*	0.22		0.3		**	90
42	00064-17-5	Ethanol	ALC	3	0.43	**0.12**	*	0.17		0.27		0.38		0.37		**	86
43	009057-02-7	Propanal	ALD	5	0.08	0		**0.21**	*	0		0		0		**	90
44	000075-15-0	Methanedithione	SDC	8	0.07	0		0		0.06		**0.18**	*	0.05		**	69
45	000071-23-8	Propan-1-ol	ALC	9	0.06	0.03		**0**	*	0.01		0.07		**0**	*	****	60
46	000123-72-8	Butanal	ALD	10	0.35	0.2		**0.13**	*	**0.12**	*	0.28		0.25		**	82
47	000078-93-3	Butan-2-one	KET	11	0.16	0.12		**0**	*	0.14		0.2		0.16		****	64
48	092112-69-1	Hexane	AHA	13	0	**0.1**	*	0.05		0.01		0.04		**0.07**	*	**	72
49	000123-73-9	But-2-enal	ALD	18	0.25	0.2		**0.11**	*	**0.09**	*	0.18		0.16		***	86
50	000590-86-3	3-Methylbutanal	ALD	19	0	0.01		0		0.01		**0.03**	*	**0.06**	*	****	60
51	068411-77-8	Cyclohexane	AHA	20	0.42	0.14		**0.03**	*	0.29		0.53		0.37		*	50
52	000110-62-3	Pentanal	ALC	22	0.04	0.07		0.07		0.08		**0.1**	*	**0.14**	*	**	90
53	000616-25-1	Pent-1-en-3-ol	ALC	23	0.1	0.1		**0.04**	*	0.07		0.12		0.08		**	78
54	017528-72-2	Pent-1-en-3-one	KET	24	0.38	0.3		**0.17**	*	**0.17**	*	0.32		0.26		*	51
55	000600-14-6	Pentane-2.3-dione	KET	25	0	**0.13**	*	0		0		0		**0.44**	*	****	85
56	003208-16-0	2-Ethylfuran	OTH	28	0.5	0.3		**0.07**	*	0.27		0.52		0.56		**	53
57	000108-10-1	4-Methylpentan-2-one	KET	29	0.04	0.02		**0**	*	**0**	*	0.03		**0.01**	*	****	50
58	068920-64-9	Methyldisulfanylmethane	SDC	35	0.33	0.22		0.22		**0.12**	*	0.33		0.54		**	94
59	000565-69-5	2-Methylpentan-3-one	KET	36	0.07	0.05		**0.03**	*	0.03		0.09		0.07		*	80
60	NID4	NID4 (LRI 734; RT 3.229) (m/z 41, 98, 69, 55, 83)	ALD	37	0	**0.02**	*	**0.01**	*	0		**0.01**	*	**0.02**	*	**	-
61	000105-46-4	Butan-2-yl acetate	ACE	40	0	0		**0.04**	*	**0.03**	*	**0.02**	*	**0.07**	*	****	72
62	025044-01-3	2-Methylpent-1-en-3-one	KET	43	0.03	0.02		0.51		0.05		0.06		**0.08**	*	*	70
63	000820-71-3	2-methylprop-2-enyl acetate	ACE	47	0	0		**0.38**	*	**0.43**	*	0.04		**0.17**	*	****	79
64	000066-25-1	Hexanal	ALD	48	19.68	19.64		8.27		**8.37**	*	15.04		13.39		*	90
65	NID2	NID2 (LRI 908; RT 8.645) (m/z 71, 41, 58, 55, 56)	NID	54	0	0		**0.1**	*	0		0		0		****	-
66	005271-38-5	2-Methylsulfanylethanol	SDC	56	0	0		0.01		**0.02**	*	0.01		**0.05**	*	****	86
67	018729-48-1	3-Methylcyclopentan-1-ol	ALC	57	0.08	0.04		**0.01**	*	0.03		0.05		0.06		*	52
68	007452-79-1	Ethyl 2-methylbutanoate	NAE	58	0.01	0.24		**0.24**	*	0		0.19		0		***	95
69	000816-11-5	Methyl 2-ethylbutanoate	NAE	59	0.01	0		0		**0.27**	*	0		0.06		**	96
70	000540-42-1	2-Methylpropyl propanoate	NAE	62	0	0.02		**0.06**	*	**0.06**	*	0.04		**0.23**	*	****	82
71	000929-22-6	(E)-Hept-4-enal	ALD	69	0.03	**0.05**	*	0		0		**0.04**	*	**0.04**	*	****	67
72	000628-63-7	Pentyl acetate	ACE	71	0	0		**0.18**	*	**0.29**	*	0.02		**0.04**	*	****	78
73	000591-23-1	3-Methylcyclohexan-1-ol	ALC	76	0.23	0.15		**0.14**	*	**0.11**	*	0.17		0.16		**	80
74	000105-68-0	1-Butanol, 3-methyl-, propanoate	NAE	77	0	0		0		0.02		0.01		**0.04**	*	***	75
75	000100-52-7	Benzaldehyde	ALD	79	0.39	0.34		**0.07**	*	0.21		0.42		0.34		****	81
76	000108-83-8	2,6-Dimethylheptan-4-one	KET	81	0.03	0.01		**0.01**	*	**0**	*	0.02		0.01		*	64
77	005441-52-1	3,5-Dimethylcyclohexan-1-ol	ALC	90	0.38	0.31		**0.19**	*	0.23		0.27		0.32		**	74
78	000104-76-7	2-Acetyloxypropyl acetate	ACE	99	1.32	2.25		2.03		1.45		1.6		**2.72**	*	***	90
79	000623-84-7	1-Acetyloxypropan-2-yl acetate	ACE	100	0.01	0.03		**0.07**	*	0.04		0.01		0.02		**	72
80	001193-81-3	(2-Methylcyclohexyl)methanol	ALC	102	0.85	0.65		0.43		**0.51**	*	0.59		0.68		*	74
81	001114-92-7 (meso)	3-Acetyloxybutan-2-yl acetate, meso	ACE	104	0.07	0.09		0.16		**0.35**	*	0.08		0.04		*	-
82	000098-86-2	1-Phenylethanone	KET	105	1.17	0.51		**0.11**	*	0.53		1.11		0.86		*	65
83	001114-92-7 (rac)	3-Acetyloxybutan-2-yl acetate, rac	ACE	107	0	0		**0.05**	*	**0.14**	*	0		0		****	88
84	000617-94-7	2-Phenylpropan-2-ol	ALC	109	2.53	2.06		**0.35**	*	1.71		1.77		1.94		***	72
85	001565-75-9	2-Phenylbutan-2-ol	ALC	111	0.33	0		**0.94**	*	0		0		0		****	72
86	000104-87-0	4-Methylbenzaldehyde	ALD	112	0	**0.12**	*	0		**0.06**	*	0.06		0.04		**	83
87	061193-21-3	Undecane	AHA	117	0.1	0.3		**0.04**	*	0.14		0.21		0.18		*	64
88	000628-66-0	3-Acetyloxypropyl acetate	ACE	120	0	0		0.02		0.07		0		0.01		****	60
89	000112-06-1	Heptyl acetate	ACE	121	0.01	0		0.02		**0.3**	*	0		0.02		***	60
90	000577-16-2	1-(2-methylphenyl)ethanone	KET	127	0.36	0.35		**0.05**	*	0.29		0.29		0.28		***	70
91	004621-04-10	4-Propan-2-ylcyclohexan-1-ol	ALC	128	0.3	0.3		0.14		0.27		**0**	*	**0.25**	*	****	70
92	000464-49-3	1,7,7-trimethylbicyclo[2.2.1]heptan-2-one	TER	129	0.98	0.66		**0.37**	*	0.58		0.75		0.8		****	98
93	31502-19-9	(E)-Non-6-en-1-ol	ALC	130	0.04	**0.19**	*	0		0		0		0		****	83
94	018829-56-6	(E)-Non-2-enal	ALD	133	0.14	**0.46**	*	0.07		0.02		0.15		**0.16**	*	**	80
95	000103-09-3	2-Ethylhexyl acetate	ACE	134	0	**0.4**	*	**0.26**	*	**0.14**	*	0		0.01		****	86
96	000557-48-2	(2E,6E)-Nona-2,6-dienal	ALD	135	0.09	**0**	*	**0**	*	**0**	*	**0**	*	0.15		***	72
97	143-08-8	Nonan-1-ol	AHA	138	0.03	0		**0.07**	*	0		0		0.06		**	79
98	002040-07-5	1-(2,4,5-Trimethylphenyl)ethanone	KET	140	0	0		**0.16**	*	0		0		0		****	83
99	93-92-5	1-Phenylethyl acetate	ACE	142	0.22	**0.05**	*	0.12		0.24		0.22		0.19		*	81
100	094094-93-6	Dodecane	AHA	143	0.03	**0.45**	*	0.03		0.07		0.05		0.08		**	87
101	000112-31-2	Decanal	ALD	144	0.49	**0.13**	*	0.26		0.27		0.32		0.45		****	97
102	000112-14-1	Octyl acetate	ACE	145	0.03	**0.47**	*	0		0.05		0		0.02		****	86
103	052844-21-0	1-Cyclohexene-1-carboxaldehyde, 2,6,6-trimethyl-	ALD	147	0	**0.07**	*	0		0		0.01		**0.05**	*	**	95
104	NID3	NID3 (LRI 1258, RT 22.147) (m/z 175, 190, 176, 57, 147)	ALD	150	0.06	0		**0.17**	*	0		0		0		**	-
105	000629-62-9	Pentadecane	AHA	159	0.07	0.14		0.01		0.02		0.01		0.07		**	96
106	000767-54-4	3,3,5-trimethylcyclohexan-1-ol	ALC	100	0.07	0.09		**0.05**	*	0.07		0.11		0.1		***	55
107	471-01-2	3,5,5-trimethylcyclohex-3-en-1-one	KET	123	0.28	0.14		0.03		0.18		0.25		0.18		*	57
108	492-37-5	2-phenylpropanoic acid	ACD	125	0.43	0.49		**0.19**	*	0.4		0.41		0.41		*	66
109	10340-23-5	(Z)-non-3-en-1-ol	ALC	135	0.15	0.08		0.27		0.27		0.28		**0.41**	*	*	81
110	74367-31-0	2-Ethyl-3-hydroxyhexyl 2-methylpropanoate	NAE	153	2.28	1.35		**0.6**	*	**0.9**	*	1.69		1.69		***	75

^a^ Non-climacteric, NC; LC or MC Light or moderate climacteric, respectively. ^b^ Order of report. Underlined numbers are compounds not interpretable in Table 4 for QTL mapping in melon chromosome 3. ^c^ Chemistry Abstract Service. ^d^ IUPAC, International Union of Pure and Applied Chemistry. ^e^ Compound classes: ACD, organic acids; ACE, acetate esters; AHA, alkanes; ALC, alcohols; ALD, aldehydes; KET, ketones; NAE, non-acetate esters; SDC, sulfur-derived compounds; TER, terpenes; OTH, others. ^f^ IDN: Identification number assigned to each volatile compound in the analysis according to its increasing order of retention time in the chromatogram. ^g^ The raw *p* values of the ANOVA were corrected for the multiple tests using the Benjamini and Hochberg (1995) false discovery rate criterion [56]. Original data were transformed for statistical analysis by applying the log transformation (base 2). ^h^ MQ: Match quality (0–100 units) of spectra compared with those of the National Institute for Standards and Technology (NIST05a.L, search version 2.0) data bank.

**Table 3:** QTL mapping of compound classes of aroma volatiles in melon chromosome 3 is depicted at the top according to [49] using the conventional nomenclature [61] for the chromosomes. For each aroma volatile, the estimated position of the QTL is indicated by a shadowed area and is colored dark when the SC [SC = ‘Shongwan Charmi’ (PI161375)] allele increased the trait or light color when it decreased the trait. Dark colors indicate values above PS, while light colors indicate values below PS levels. *ETHQB3.5* is the region containing the QTL of the climacteric. I/D: Ethylene dependence (1) and independence (−1).

									Molecular Marker Position																	
Physical Distance in the Genetic Map		20,373,958	20,816,308	24,346,383	24,668,302	24,780,842	24,926,308	24,931,945		25,197,968		26,205,074	26,328,662		26,434,021	26,788,316	26,801,772		27,143,907	27,145,624						
**Compound Classes**		**CMPSNP556**	**A_21-C11**	**ECM208**	**mc296EST**	**CMPSNP8**	**CMCTN5**	**CMN22-85**		**CMPSNP374**		**ECM60c**	**AI_06_G01**		**AI_14-F04**	**ECM205**	**MC215**		**TJ10**	**ECM125**	N Confirmed > PS	N Confirmed < PS	N QTLclim > PS	N QTLclim < PS	Significance (*p* < 0.05)	Ethylene I/D
Acetate Esters																					1	−	1	−	****	1
Sulfur Derived Compounds																					1	−	1	−	****	1
Aldehydes																					−	1	−	−	***	−1
Ketones																					−	1	−	−	****	−1
	Climacteric QTL position (*ETHQB3.5*)																									

**Table 4:** QTL mapping of individual aroma volatile compounds in melon chromosome 3 is depicted at the top according to [49] using the conventional nomenclature [61] for the chromosomes. For each aroma volatile, the estimated position of the QTL is indicated by a shadowed area (grey or black) when the SC [‘Shongwan Charmi’ (PI161375)] allele increased or decreased the trait, respectively. Question mark indicates position of mapping in doubt.

	**IUPAC ^a^ Name of Aroma Volatile Compounds**		**CMPSNP556**	**A_21−C11**	**ECM208**	**mc296EST**	**CMPSNP8**	**CMCTN5**	**CMN22−85**		**CMPSNP374**		**ECM60c**	**AI_06−G01**		**AI_14−F04**	**ECM205**	**MC215**		**TJ10**	**ECM125**		N QTL Confirmed > PS	N QTL Confirmed < PS	N QTL in ETHQB3.5 Region > PS	N QTL in ETHQB3.5 Region < PS	CAS Number ^b^	Ethylene I/D ^c^
												*ETHQB3.5*													Colocalization with *ETHQB3.5*			
**IDN ^d^ Climacteric QTL**																												
**position**																												
1	Methyl propanoate																						1	−	1	−	000554-12-1	1
2	1-Methylethyl acetate																						1	−	1	−	000108-21-4	1
3	S-Methylmet ethanethioate																						1	−	−	−	001534-08-3	−1
5	Methyl butanoate																						1	−	1	−	000623-42-7	1
6	2-Methylbutan-1-ol																						1	−	1	−	000137-32-6	1
7	2-methylpropyl acetate																						1	−	1	−	000110-19-0	1
8	Methyl 2-methylbutanoate																						1	−	−	−	000868-57-5	−1
10	Propyl propanoate																						1	−	1	−	000106-36-5	1
11	Butyl acetate																						1	−	1	−	000123-86-4	1
12	1-Methylsulfanylbutan-1-one																						1	−	1	−	002432-51-1	1
13	3-Methylbutyl acetate																						1	−	1	−	000123-92-2	1
14	2-Methylbutyl acetate																						1	−	−	−	000624-41-9	−1
15	Ethenylbenzene																						−	1	−	1	000100-42-5	1
16	Methyl 2-methylsulfanylacetate																						1	−	1	−	016630-66-3	1
17	3-Methylbut-2-enyl acetate																						1	−	−	−	001191-16-8	−1
18	3-Methyl-1-methylsulfanyl-butan-1-one																						1	−	1	−	023747-45-7	1
20	1-(3-Hydroxypropylsulfanyl)ethanone																						1	−	1	−	115051-66-6	1
21	Hexyl acetate																						1	−	−	−	000142-92-7	−1
22	1,8,8-Trimethyl-7-oxabicyclo[2-2-2]octane																						1	−	1	−	000470-82-6	1
24	NID1 (LRI 1143; RT 20.871) (*m*/*z* 43, 88, 73, 61, 148, 41, 45)																						1	−	1	−	NID1	1
26	Benzyl acetate																						1	−	1	−	000140-11-4	1
30	2-Phenylethyl acetate																						1	−	−	−	000103-45-7	−1
31	(1-Hydroxy-2,4,4-trimethyl-pentan-3-yl) 2-methylpropanoate																						−	1	−	−	074367-33-2	−1
39	(E)-4-(2,6,6-Trimethyl-1-cyclohexenyl)but-3-en-2-one																						1	−	−	−	000079-77-6	−1
41	Methanethiol																						−	1	−	−	000074-93-1	−1
46	Butanal																						−	1	−	−	000123-72-8	−1
49	But-2-enal																						−	1	−	−	000123-73-9	−1
54	Pent-1-en-3-one																						−	1	−	−	017528-72-2	−1
57	4-Methylpentan-2-one																						−	1	−	1	000108-10-1	1
58	Methyldisulfanylmethane																						−	1	−	−	068920-64-9	−1
60	NID4 (LRI 734; RT 3.229) (*m*/*z* 41, 98, 69, 55, 83)																						1	−	−	−	NID4	−1
61	Butan-2-yl acetate																						1	−	1	−	000105-46-4	1
63	2-methylprop-2-enyl acetate																						1	−	1	−	000820-71-3	1
64	Hexanal																						−	1	−	−	000066-25-1	−1
69	Methyl 2-ethylbutanoate																						1	−	−	−	000816-11-5	−1
70	2-Methylpropyl propanoate																						1	−	1	−	000540-42-1	1
72	Pentyl acetate																						1	−	1	−	000628-63-7	1
73	3-Methylcyclohexan-1-ol																						−	1	−	−	000591-23-1	−1
76	2,6-Dimethylheptan-4-one																						−	1	−	−	000108-83-8	−1
80	(2-Methylcyclohexyl)methanol																						−	1	−	−	001193-81-3	−1
81	3-Acetyloxybutan-2-yl acetate, meso																						1	−	−	−	001114-92-7 (meso)	−1
83	3-Acetyloxybutan-2-yl acetate, rac																						1	−	−	−	001114-92-7 (rac)	−1
89	Heptyl acetate																						1	−	−	−	000112-06-1	−1
110	2-Ethyl-3-hydroxyhexyl 2-methylpropanoate																						−	1	−	−	74367-31-0	−1
	Total QTLs																						31	13	19	2		

Abbreviations: NID, unidentified compound. ^a^ IUPAC, International Union of Pure and Applied Chemistry. ^b^ Chemical Abstracts Service. ^c^ Numbers indicate the ethylene dependence classification of QTLs. (−1) Fully independent. (1) Fully dependent. ^d^ Identification number is assigned to each volatile compound in the analysis according to its increasing order of retention time in the chromatogram (as in Table 2).

**Table 5:** Groups with at least two compounds and correlations above 0.7 in absolute value were obtained by correlation network analysis (CNA) applied to melon aroma volatile compounds. The chemical and sensory attributes of the compounds are in Table S6.

Order ^a^	CAS ^b^ and Group	IUPAC ^c^ Name
	Group 1	
1	000079-20-9	Methyl acetate
2	000078-83-1	2-Methylpropan-1-ol
3	000554-12-1	Methyl propanoate
4	001534-08-3	S-Methyl ethanethioate
5	000109-60-4	Propyl acetate
6	000623-42-7	Methyl butanoate
7	000137-32-6	2-Methylbutan-1-ol
8	000110-19-0	2-Methylpropyl acetate
9	000868-57-5	Methyl 2-methylbutanoate
10	000820-71-3	2-Methylprop-2-enyl acetate
11	000106-36-5	Propyl propanoate
12	000123-86-4	Butyl acetate
13	002432-51-1	1-Methylsulfanylbutan-1-one
14	005271-38-5	2-Methylsulfanylethanol
15	000540-42-1	2-Methylpropyl propanoate
16	000123-92-2	3-Methylbutyl acetate
17	000624-41-9	2-Methylbutyl acetate
18	000628-63-7	Pentyl acetate
19	016630-66-3	Methyl 2-methylsulfanylacetate
20	001191-16-8	3-Methylbut-2-enyl acetate
21	023747-45-7	3-Methyl-1-methylsulfanyl-butan-1-one
22	115051-66-6	1-(3-Hydroxypropylsulfanyl)ethanone
23	000142-92-7	Hexyl acetate
24	000470-82-6	1,8,8-Trimethyl-7-oxabicyclo[2-2-2]octane
25	NID1 ^d^	NID1 (LRI 1143; RT 20.871) (*m*/*z* 43, 88, 73, 61, 148, 41, 45)
26	000140-11-4	Benzyl acetate
27	007371-86-0	4-Acetyloxypentan-2-yl acetate
28	000103-45-7	2-Phenylethyl acetate
	Group 2	
1	000123-72-8	Butanal
2	000123-73-9	But-2-enal
3	000616-25-1	Pent-1-en-3-ol
4	017528-72-2	Pent-1-en-3-one
5	000565-69-5	2-Methylpentan-3-one
6	000066-25-1	Hexanal
	Group 3	
1	068411-77-8	Cyclohexane
2	000108-88-3	Methylbenzene
3	018729-48-1	3-Methylcyclopentan-1-ol
4	000105-54-4	Ethyl butanoate
	Group 4	
1	NID2 ^d^	NID2 (LRI 908; RT 8.645) (*m*/*z* 71, 41, 58, 55, 56)
2	001565-75-9	2-Phenylbutan-2-ol
3	002040-07-5	1-(2,4,5-Trimethylphenyl)ethanone
	Group 5	
1	000100-42-5	Ethenylbenzene
2	000591-23-1	3-Methylcyclohexan-1-ol
3	005441-52-1	3,5-Dimethylcyclohexan-1-ol
4	001193-81-3	(2-Methylcyclohexyl)methanol
5	000464-49-3	1,7,7-Trimethylnorbornan-2-one
6	074367-33-2	(1-Hydroxy-2,4,4-trimethyl-pentan-3-yl) 2-methylpropanoate
7	74367-31-0	2-Ethyl-3-hydroxyhexyl 2-methylpropanoate

^a^ Arbitrary order within each group according to correlation network analysis. Underlined numbers are compounds not interpretable in Table 4 for QTL mapping in melon chromosome 3. ^b^ CAS, Chemical Abstracts Service (number of compounds). ^c^ IUPAC, International Union of Pure and Applied Chemistry. ^d^ All unidentified compounds are identified with linear retention index, retention time (min), and main ions.

## Data Availability

The data used to support the findings of this study can be made available by the corresponding author upon request.

## Supplementary Materials

The following supporting information can be downloaded at: https://www.mdpi.com/article/10.3390/foods12020376/s1, Table S1. A list of molecular markers was used in this study and is located in MELOGEN v4.0; Table S2. The melon graphical genotype of the NILs was used in this study; Table S3. A list of genes, descriptions, and properties was obtained from the MELOGEN database v4.0 that were covered by molecular markers in the region of the QTL of the climacteric *ETHQB3.5*, and the introgressions were covered by this experiment; Table S4 [97,98,99,100,101,102,103,104,105,106,107,108,109,110,111,112,113,114,115,116,117,118,119,120,121,122,123,124,125,126,127,128,129,130,131,132,133,134,135,136,137,138,139,140,141,142,143,144,145,146,147,148,149,150,151,152,153,154,155,156,157,158,159,160,161,162,163,164,165,166,167,168,169,170,171,172,173,174,175,176]. Chemical and sensory attributes of the main melon fruit volatile compounds were used for QTL mapping in melon chromosome 3, identified in the five melon Near-Isogenic Lines (NILs) and the parental control ‘Piel de Sapo’ (PS).; Table S5. Main fruit volatile compounds were identified for QTL mapping on melon chromosome 3 identified in the headspace of the fruit of the non-climacteric Near-Isogenic Line SC3-5-7 and the parental line ‘Piel de Sapo’ (PS); Table S6. Chemical and sensory attributes of the most numerous grouping of the individual volatile compounds were obtained in the correlation network analysis applied to Near-Isogenic Lines (NILs) with introgression on melon chromosome 3 and the parental control ‘Piel de Sapo’ (PS); Figure S1. Respiration rate (RR) and ethylene production (EP) were obtained via Near-Isogenic Line SC3-5-13 (mean ± SE, *n* = 3) during postharvest ripening at 21 °C and relative humidity of 66 ± 6% for 10 days (season 2).
