# Supplementary material for: Thorough Characterization of ETHQB3.5, a QTL Involved in Melon Fruit Climacteric Behavior and Aroma Volatile Composition
Source: Foods. 2023 Jan 13;12(2):376. doi: 10.3390/foods12020376 (PMC9858179; doi:10.3390/foods12020376)
Supplement: Supplementary file 1 [file foods-12-00376-s001.zip › Dos-Santos Figure S1. RR EP SC3-5-13 final2.pdf]

Supplementary Figure S1

Thorough characterization of *ETHQB3.5*, a QTL involved in climacteric behavior and aroma volatile composition located on chromosome 3 of melon genome

Noelia Dos-Santos, María C. Bueso, Aurora Díaz, Eduard Moreno,
Jordi Garcia-Mas, Antonio J. Monforte, J. Pablo Fernández-Trujillo

Foods-MDPI

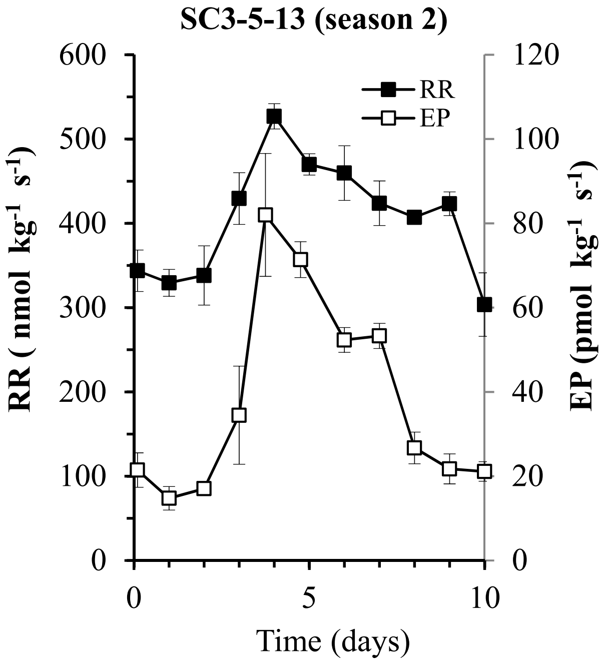

Supplementary Figure S1. Respiration rate (RR) and ethylene production (EP) in Near-Isogenic Line SC3-5-13 (mean ± SE, n=3) during postharvest ripening at 21 ºC and relative humidity of 66±6% during 10 days (season 2).
